# Association of intraocular lens tilt and decentration with visual acuity using SS-OCT-based analysis

**DOI:** 10.1007/s00417-025-06965-9

**Published:** 2025-10-16

**Authors:** Joukje C. Wanten, Noël J. C. Bauer, Alle Boonstra, Tos T. J. M. Berendschot, Rudy M. M. A. Nuijts

**Affiliations:** University Eye Clinic Maastricht, Maastricht University Medical Center+, P.O. Box 5800, Maastricht, 6202 AZ the Netherlands

**Keywords:** Tilt, Decentration, Intraocular lens, Visual acuity, Optical coherence tomography, Cataract surgery

## Abstract

**Purpose:**

To evaluate the association of postoperative IOL tilt and decentration and corrected distance visual acuity (CDVA) by performing analyses using IOLMaster700 data and custom-developed software.

**Setting:**

University Eye Clinic Maastricht, Maastricht University Medical Center, Maastricht, The Netherlands.

**Methods:**

This case series included patients after cataract surgery who underwent SS-OCT scans using the IOLMaster700 with custom-developed software for tilt and decentration analyses. The outcomes based on our custom-developed software were conducted along the pupillary axis (PA) and corneal topographic axis (CTA). Associations between tilt, decentration and CDVA were evaluated.

**Results:**

A total of 168 eyes (168 patients) were analyzed. The maximum observed IOL tilt was 9.54° along the CTA and 3.69° along the PA. Maximum decentration reached 2.22 mm (CTA) and 2.17 mm (PA). Mean tilt was 1.21 ± 0.69° (PA) and 4.87 ± 1.52° (CTA), while mean decentration was 0.27 ± 0.25 mm (PA) and 0.27 ± 0.26 mm (CTA). Regression analyses showed no statistically significant associations between tilt or decentration and CDVA, irrespective of the reference axis used. For tilt along the PA, the regression coefficient (B) was 0.004 (95%CI -0.011, 0.020; *P* = 0.592), for tilt along the CTA, B was − 0.006 (95%CI -0.013, 0.001; *P* = 0.082). For decentration, B was 0.012 (95%CI -0.020, 0.044; *P* = 0.455) and 0.002 (SE = 0.018, 95%CI -0.034, 0.037; *P* = 0.925) along the PA and CTA, respectively.

**Conclusion:**

In this cohort, IOL tilt and decentration were generally small, with no detectable association with CDVA. The findings emphasize that different assessment methods should not be used interchangeably, and potential effects cannot be excluded given the limited sample and range of values.

**Supplementary Information:**

The online version contains supplementary material available at 10.1007/s00417-025-06965-9.

## Key messages

***What is known?***

- Intraocular lens (IOL) tilt and decentration are generally seen as risk factors for suboptimal visual acuity following cataract surgery.
- Accurately measuring IOL tilt and decentration is challenging due to the lack of a universal reference axis.

***What is new?***

- Using SS-OCT and custom software, IOL tilt and decentration were generally small, with no detectable association with corrected distance visual acuity (CDVA) in this cohort.
- IOL tilt and decentration analyses along the pupillary axis (PA) and cornea topographic axis (CTA) showed outcome differences, highlighting different methods of analyses cannot be used interchangeably.

**Supplementary Information:**

The online version contains supplementary material available at 10.1007/s00417-025-06965-9.

## Introduction

Intraocular lens (IOL) tilt and decentration are significant risk factors for suboptimal visual acuity outcomes after cataract surgery in healthy eyes [1]. Tilt refers to the angle between the IOL plane and a reference axis, while decentration indicates the distance between them [2, 3]. Commonly reported tilt and decentration outcomes, ranging from 2–3⁰ and 0.2–0.3 mm, often go unnoticed by patients [1]. Research suggests that tilt exceeding 5–7⁰ or decentration over 0.4–0.5 mm are clinically significant, as they can induce optical aberrations that adversely affect IOL performance [4–8]. However, most of these thresholds are derived from model analyses, and real-world tolerances may vary depending on IOL design [5–8]. Certain designs, such as toric and multifocal IOLs (mIOLs) appear more susceptible to tilt and decentration, which can result in less predictable astigmatism outcomes and altered light distributions between focal points [9–13]. Despite the established clinical significance of tilt and decentration, the exact impact on visual acuity remains controversial in the literature [1, 12, 14, 15]. 

**Fig. 1:**
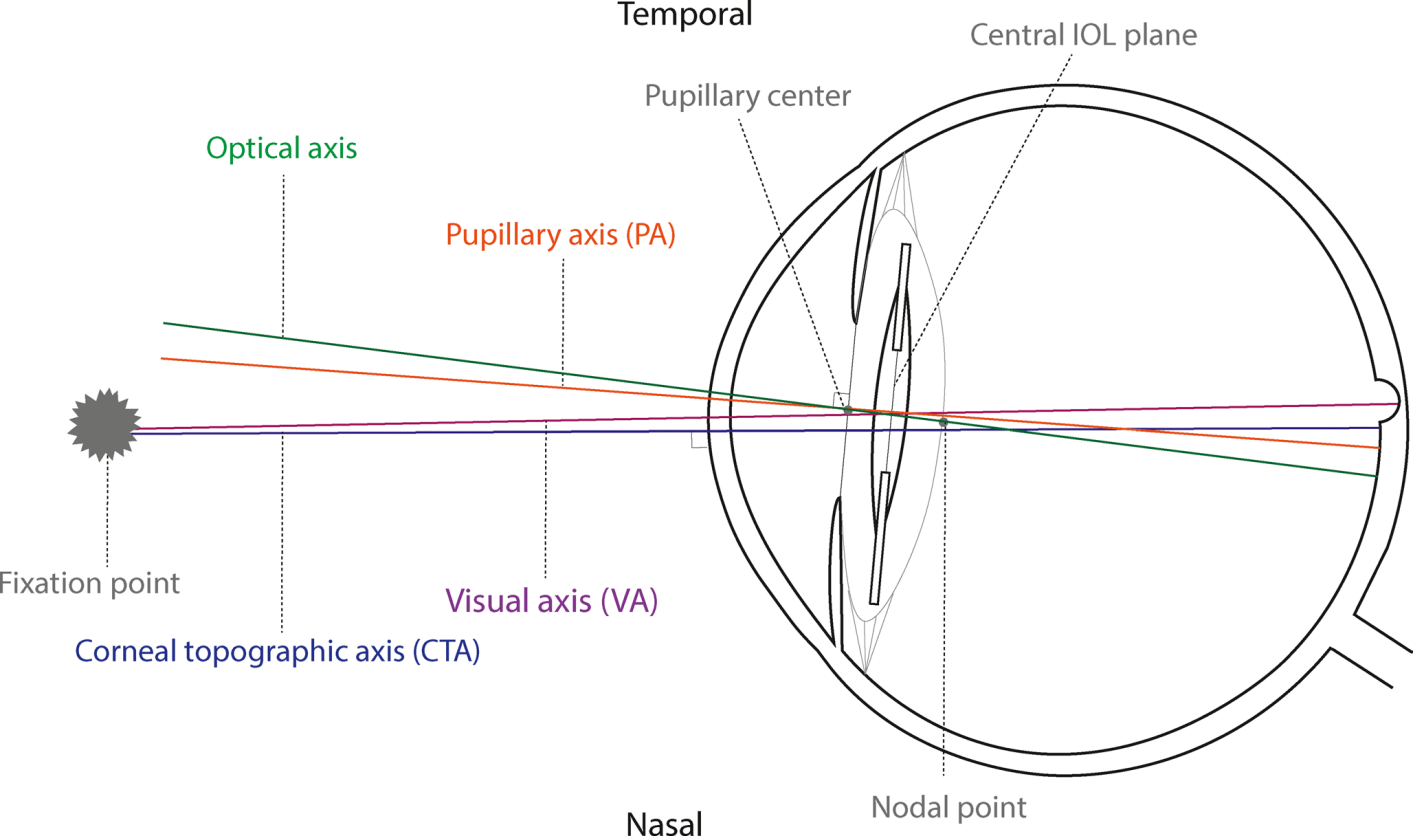
Schematic overview of different ocular reference axes, including the optical axis (green), pupillary axis (orange), corneal topographic axis (blue), and visual axis (purple). Angle alpha (**α**) is defined as the angle between the optical and visual axis. Angle kappa (κ) is defined as the angle between the pupillary and visual axis. Abbreviations: CTA (Corneal Topographic Axis), PA (Pupillary Axis), VA (Visual Axis)

Accurately measuring IOL tilt and decentration postoperatively is challenging due to the lack of a universal reference axis. During cataract surgery, the IOL is usually centered by aligning the IOL with the patient’s visual axis using the Purkinje reflex. The visual axis is defined as the axis which connects the fixation point with the fovea through the nodal points of the eye, while the optical axis connects the three Purkinje images. However, for postoperative assessment of IOL tilt and decentration, devices such as the IOLMaster700 (Carl Zeiss Meditec, Jena, Germany) that utilize Swept-Source Optical Coherence Tomography (SS-OCT) rely on alternative reference axes, as they cannot precisely determine the visual axis [16, 17]. This absence of a universal reference axis has led to the use of different axes in tilt and decentration analyses, raising concerns regarding the comparability of results across studies. Figure 1 shows the axes which are commonly used in practice [1, 15, 16, 18]. The pupillary axis (PA) extends from the corneal surface through the pupillary center, and the corneal topographic axis (CTA) is a perpendicular line through the fixation point that intersects the anterior corneal surface at the corneal vertex [19].

This study aims to evaluate the association between IOL tilt and decentration and corrected distance visual acuity (CDVA), using IOLMaster700 data analyzed with custom-developed software. These analyses are conducted along different reference axes to assess their clinical implications. Furthermore, the study offers insight into the range and potential clinical thresholds of postoperative IOL tilt and decentration observed in routine cataract surgery.

## Methods

This is a single-center validation and descriptive study in the University Eye Clinic Maastricht of the Maastricht University Medical Centre (MUMC+), the Netherlands. From December 2021 until April 2023 data was collected from patients who underwent cataract surgery on the right eye. The study was approved by the local medical ethics committee and executed in accordance with the principles of the Helsinki Declaration.

### Data collection

Patients were included if they were ≥ 18 years and had finished their last postoperative visit after cataract surgery. Exclusion criteria encompassed sight-threatening comorbidities, cornea opacities, conditions that hindered the patient’s ability to focus on the fixation target during the measurements, and poor image quality affecting tilt and decentration analyses. The following variables were collected from electronic medical records: demographics, medical history, surgery data, relevant biometry values, visual acuities, refraction, and IOL design. All eyes included in the study underwent postoperative undilated SS-OCT imaging using the IOLMaster700 with fixation tracked through the device’s visualization of the fovea centralis. Consecutive scans were captured from six different angles (0°, 30°, 60°, 90°, 120° and 150°).

CDVA was assessed using either a Snellen chart or an Early Treatment Diabetic Retinopathy Study (ETDRS) chart at 4 m. The last correctly identified line on the chart was recorded, continuing until no further optotypes could be discerned.

### Custom-developed software for tilt and decentration analyses

Lens tilt and decentration analyses were performed by custom-developed software using MATLAB (Version 2021b, The Mathworks, Natick, United States of America). Prior to analysis, images were cropped while retaining anterior segment data, underwent noise removal, applying non-local mean denoising, and then underwent sharpening and contrast enhancements [20]. Binary segmentation techniques were employed to identify the cornea, iris, and lens.

To ensure transparency and reproducibility, all data preparation steps were implemented using standardized procedures embedded within the MATLAB code. A detailed description of the software workflow, including data preparation and image processing, is provided in the software manual in Appendix 1.For a 3D-reconstruction of the IOL, the pupillary center was determined in the six different angles, using the upper and lower halves of the iris in the images. Parabolas were then fitted along the anterior and posterior lens surfaces, defined by the formula: $\:a{(x-{x}_{0})}^{2}+c$. To achieve optimal fitting for the IOL thickness, multiple weighted parabolas were utilized and incorporated into a minimized cost function. The parabola with parameter $\:c$ represented the most optimal fit and carried the highest weight. Any deviation to the left ($\:c$−1 and so on) augmented the score, indicating a poorer fit, while deviations to the right ($\:c$+1 and so on) diminished the score, albeit to a lesser extent than the original parabola due to the assigned weight. While toric IOLs deviate from a perfect parabolic surface, this approximation was used to determine lens positioning (tilt and decentration) rather than to capture astigmatic optics. Any systematic error introduced by this simplification is expected to be smaller than the known measurement variability of SS-OCT devices and below thresholds considered clinically relevant.

**Fig. 2:**
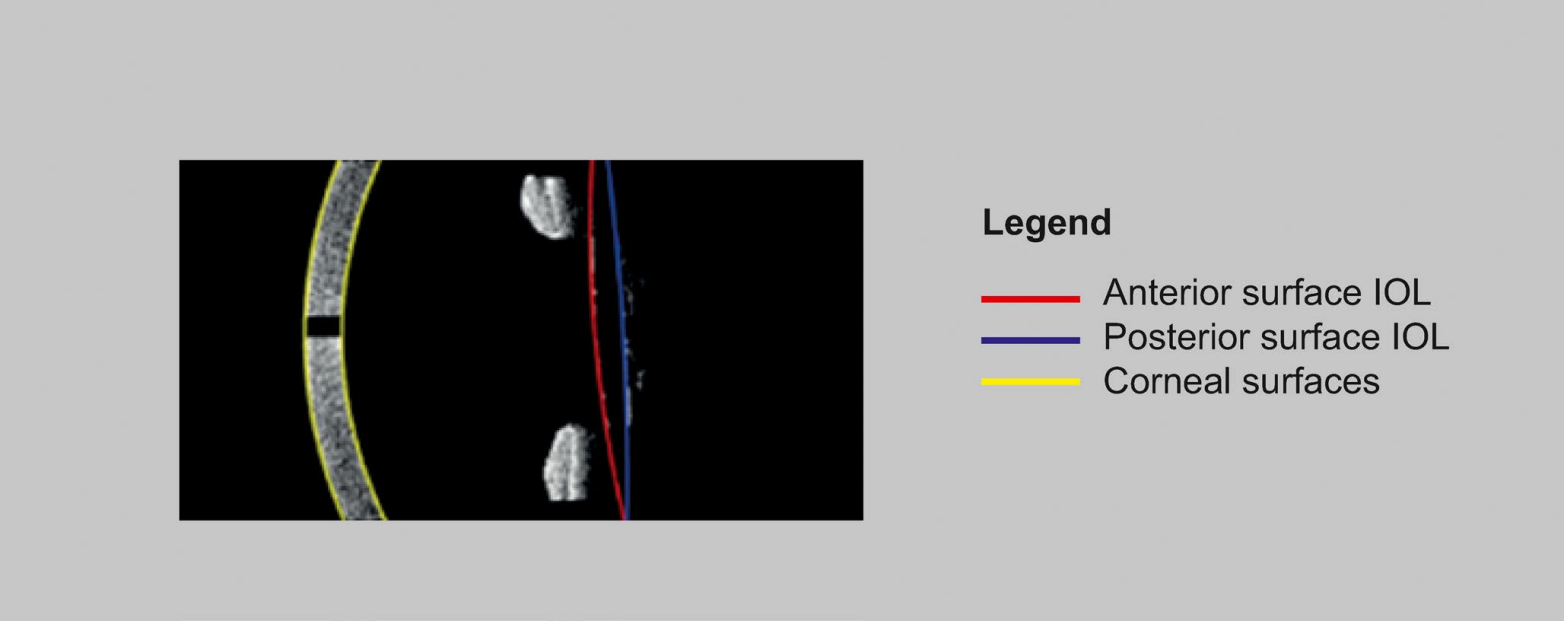
Tilt and decentration analysis using our custom-developed software and IOLMaster700 images. This software identifies the corneal surfaces and anterior and posterior lens surface to assess the tilt and decentration

The analysis of lens tilt entailed the calculation of connecting lines between the parabola intersections for each angle-slice. A central plane was then constructed employing 100 data points for each line, and the normal vector of this plane, in comparison to the reference axis, determined the lens tilt. To assess lens decentration, the center of the lens was used, presuming it to be its thickest point. The thickness, defined as the distance between the anterior and posterior surfaces perpendicular to the line passing through the center of the lens, was calculated at 100 evenly distributed points along this line. A parabola was fitted for each angle in each scan. The thickest point was identified by fitting a paraboloid to these data points using the formula: $\:a\left({\left(x-{x}_{0}\right)}^{2}+{\left(y-{y}_{0}\right)}^{2}\right)+c$. The thickest point, denoted as ($\:{x}_{0},{y}_{0},c$), was then utilized to determine lens decentration by comparing intersections of the reference axis with the central lens plane. The difference in x- and y-coordinates between these intersections and the thickest point delineated the lens’ decentration relative to the reference axis. Figure 2 shows an angle-slice, with the corneal and lens surfaces identified using our custom-developed software for tilt and decentration assessment.

As in clinical practice, tilt and decentration are commonly evaluated with respect to the CTA and PA, and therefore both reference axes are used in our analyses. To ascertain the CTA, the vertex of the anterior cornea was identified using the $\:{x}_{0}$ and $\:c$ parameters. This axis was established by fitting a paraboloid through the vertex of the anterior corneal parabolas, followed by determination of the tangent plane. The PA was computed by establishing the line connecting the centers of the iris from each slice. Tilt and decentration were then calculated using the normal vectors of both planes of the reference axes. We assumed that the precise direction of tilt and/or decentration did not affect our results, given the exploratory nature of this study.

#### Statistical analysis

This study was exploratory in nature, and the sample size was based on all eligible patients who met the inclusion criteria during the study period. No a priori power analysis was performed, as the primary aim was to describe postoperative IOL tilt and decentration in a real-world clinical cohort and to explore potential associations with CDVA.

All statistical analyses were performed using SPSS (IBM Corp. Released 2021. IBM SPSS Statistics for Windows, Version 28.0. Armonk, NY). Frequencies and descriptive statistics were employed for the analysis of quantitative variables, including mean and standard deviation (SD). Visual acuity outcomes, originally obtained using either Snellen or ETDRS charts, were converted to logMAR units using standard conversion formulas prior to analysis.

Associations between IOL tilt or decentration and ocular or surgical factors were first assessed using univariable linear regression. Variables identified as potentially relevant, either clinically or based on a significance level were subsequently included in multivariable linear regression models using the forced entry method. To evaluate the robustness of these models, a backward stepwise regression analysis was also conducted as a sensitivity check.

In case of non-normal distribution, a logarithmic transformation was applied before conducting the regression analyses and model assumptions were carefully assessed. The distribution of residuals was visually inspected using Q-Q plots and histograms to confirm approximate normality. To assess whether the relationship between CDVA and either IOL tilt or decentration differed by IOL type (monofocal or EDOF), additional multivariable regression models were performed including interaction terms (tilt*IOL type or decentration*IOL type), adjusted for age. During model development, lens thickness and IOL power showed high correlation with anterior chamber depth (ACD) and axial length (AL), respectively, and were excluded from the final models to reduce collinearity. The quantification of decentration, and angles alpha and kappa involved the determination of their horizontal and vertical components. The magnitudes of these variables were calculated using the Euclidean distance, which is obtained by taking the square root of the sum of the squares of their respective horizontal and vertical components. A *P* ≤ 0.05 was considered statistically significant.

## Results

Table 1 presents the baseline characteristics of the 168 operated right eyes from 168 patients (82 males, 86 females) included in this study. Patients received either a monofocal intraocular lens (IOL, *n* = 118) or an extended depth-of-focus (EDOF) IOL (*n* = 50). The implanted IOLs included the AcrySof^®^ IQ Monofocal IOL (Alcon, Fort Worth, TX, USA), AcrySof^®^ IQ Vivity IOL (Alcon, Fort Worth, TX, USA), and Acunex^®^ Vario IOL (Teleon Surgical BV, Spankeren, the Netherlands). Both toric and non-toric IOLs were used. Eleven patients underwent an intraocular procedure prior to cataract surgery, with pars plana vitrectomy (PPV) performed in 10 eyes and trabeculectomy in one eye. Six patients underwent cataract surgery combined with either an iStent (Glaukos Corp., Laguna Hills, California, United States of America) implantation for primary open angle glaucoma (*n* = 5) or a PPV for a macular pucker (*n* = 1). Intraoperative complications included three posterior capsular ruptures and one irregular, but continuous curvilinear capsulorhexis, considered relevant factors for post-surgery IOL tilt or decentration.

**Table 1:** Baseline characteristics

Variable	Mean ± SD	Range
Age (years)	71 ± 11	17; 92
CDVA (logMAR)	0.01 ± 0.07	−0.18; 0.28
SEQ (D)	−0.15 ± 0.62	−3.50; 1.25
Absolute prediction error (D)	0.32 ± 0.30	0.00; 1.63
AL (mm)	24.1 ± 1.5	21.6; 30.2
ACD (mm)	3.16 ± 0.40	2.00; 4.01
WTW (mm)	12.1 ± 0.39	11.1 ± 13.1
Angle alpha (mm)	0.48 ± 0.15	0.10; 1.08
Angle kappa (mm)	0.34 ± 0.16	0.01; 0.92

*ACD* (Anterior Chamber Depth), *AL* (Axial Length), *CDVA* (Corrected Distance Visual Acuity), *D* (Diopter), *SD* (Standard Deviation), *SEQ* (Spherical Equivalent), *WTW* (white−to−white)

## Tilt and decentration along different reference axes

The tilt (*n* = 168) and decentration (*n* = 162) analyses conducted using IOLMaster700 images and our custom-developed software employed different reference axes, including the CTA and PA. Mean tilt outcomes in this cohort along the PA were 1.21 ± 0.69° (range 0.09; 3.69), and along the CTA 4.87 ± 1.52° (range 0.25; 9.54). The decentration outcomes along the PA had a mean value of 0.27 ± 0.25 mm (range 0.02; 2.17), and 0.27 ± 0.26 mm (range 0.02; 2.22) along the CTA.

### Associated factors for tilt and decentration

Univariable and multivariable linear regression analyses identified variables associated with tilt and decentration along both reference axes, as shown in Tables 2 and 3. For tilt along the PA, statistically significant associations were found with preoperative angle alpha and combined surgery. For tilt along the CTA, axial length (AL), preoperative angles alpha and kappa, and intraoperative complications emerged as significantly associated factors. Decentration measured along both axes showed a statistically significant association with AL. Additionally, backward stepwise regression, conducted as a sensitivity analysis, confirmed these findings and identified age and intraoperative complications as significantly associated factors with tilt along the PA, complementing those identified in the forced entry analysis.

**Table 2:** Univariable and multivariable linear regression analyses for both Tilt and decentration along the pupillary axis

	Tilt					Decentration				
	Univariable linear regression		Multivariable linear regression			Univariable linear regression		Multivariable linear regression		
Variable	Unstandardized B-coefficient (SE)	*P*	Unstandardized B-coefficient (SE)	95%CI	*P*	Unstandardized B-coefficient (SE)	*P*	Unstandardized B-coefficient (SE)	95%CI	*P*
Age (year)	0.008 (0.005)	0.113	0.009 (0.005)	−0.001; 0.020	0.087	0.001 (0.003)	0.814	0.003 (0.003)	−0.003; 0.009	0.269
Female sex	0.162 (0.106)	0.128	0.097 (0.110)	−0.120; 0.315	0.377	−0.095 (0.055)	0.086	−0.032 (0.060)	−0.150; 0.086	0.589
AL (mm)	−0.109 (0.035)	0.002	−0.019 (0.046)	−0.109; 0.071	0.684	0.079 (0.018)	< 0.001	0.087 (0.026)	0.036; 0.138	< 0.001
ACD (mm)	−0.312 (0.133)	0.020	0.002 (0.160)	−0.313; 0.318	0.990	0.176 (0.068)	0.011	0.009 (0.086)	−0.160; 0.178	0.919
WTW (mm)	−0.158 (0.141)	0.263	0.034 (0.151)	−0.266; 0.333	0.825	0.108 (0.072)	0.134	0.010 (0.083)	−0.153; 0.173	0.906
Angle alpha (mm)	1.273 (0.353)	< 0.001	1.290 (0.375)	0.549; 2.031	< 0.001	−0.466 (0.186)	0.013	−0.138 (0.202)	−0.534; 0.258	0.497
Angle kappa (mm)	0.296 (0.333)	0.408	−0.029 (0.327)	−0.676; 0.618	0.930	−0.300 (0.177)	0.093	−0.164 (0.180)	−0.517; 0.189	0.366
Junior surgeon (yes)	0.136 (0.114)	0.234	0.103 (0.116)	−0.126; 0.333	0.375	0.047 (0.058)	0.419	0.049 (0.062)	−0.073; 0.171	0.432
History of intraocular surgery (yes)	−0.559 (0.211)	0.009	−0.343 (0.237)	−0.811; 0.126	0.150	0.021 (0.111)	0.848	−0.016 (0.125)	−0.261; 0.229	0.901
Underwent combined surgery (yes)	−0.665 (0.283)	0.020	−0.839 (0.295)	−1.422; −0.256	0.005	−0.050 (0.145)	0.732	−0.083 (0.155)	−0.387; 0.221	0.592
Intraoperative complication (yes)	0.557 (0.348)	0.111	0.524 (0.345)	−0.159; 1.206	0.131	0.061 (0.177)	0.732	0.205 (0.182)	−0.152; 0.562	0.263

*ACD* anterior chamber Depth, *AL* axial length, *PA* pupillary axis, *SE* standard error, *WTW* white−to−white

**Table 3:** Univariable and multivariable linear regression analyses for both Tilt and decentration along the corneal topographic axis (CTA). Decentration outcomes were log-transformed

	Tilt					Decentration				
	Univariable linear regression		Multivariable linear regression			Univariable linear regression		Multivariable linear regression		
Variable	Unstandardized B-coefficient (SE)	*P*	Unstandardized B-coefficient (SE)	95%CI	*P*	Unstandardized B-coefficient (SE)	*P*	Unstandardized B-coefficient (SE)	95%CI	*P*
Age (year)	0.002 (0.011)	0.888	−0.004 (0.009)	−0.022; 0.014	0.722	−0.005 (0.002)	0.025	−0.003 (0.003)	−0.009; 0.003	0.253
Female sex	0.126 (0.235)	0.593	0.003 (0.180)	−0.351; 0.357	0.985	−0.029 (0.051)	0.563	0.003 (0.054)	−0.103; 0.109	0.956
AL (mm)	−0.465 (0.069)	< 0.001	−0.358 (0.075)	−0.504; −0.212	< 0.001	0.067 (0.016)	< 0.001	0.068 (0.023)	0.023; 0.113	0.004
ACD (mm)	−0.792 (0.281)	0.005	0.397 (0.261)	−0.115; 0.909	0.130	0.131 (0.062)	0.036	−0.027 (0.078)	−0.180; 0.126	0.726
WTW (mm)	−0.474 (0.297)	0.113	0.057 (0.247)	−0.427; 0.541	0.817	0.134 (0.064)	0.039	0.043 (0.075)	−0.104; 0.190	0.569
Angle alpha (mm)	6.188 (0.608)	< 0.001	5.093 (0.613)	3.890; 6.296	< 0.001	−0.368 (0.168)	0.030	−0.144 (0.183)	−0.504; 0.216	0.433
Angle kappa (mm)	2.828 (0.689)	< 0.001	1.524 (0.535)	0.476; 2.572	0.005	−0.078 (0.160)	0.625	0.008 (0.163)	−0.312; 0.328	0.962
Junior surgeon (yes)	0.136 (0.244)	0.577	0.078 (0.190)	−0.294; 0.450	0.682	−0.012 (0.053)	0.825	0.027 (0.056)	−0.083; 0.137	0.634
History of intraocular surgery (yes)	−0.144 (0.475)	0.763	0.310 (0.387)	−0.447; 1.067	0.425	0.033 (0.100)	0.741	0.030 (0.114)	−0.193; 0.253	0.795
Underwent combined surgery (yes)	0.717 (0.613)	0.244	−0.083 (0.482)	−1.028; 0.862	0.864	−0.169 (0.131)	0.199	−0.092 (0.141)	−0.369; 0.185	0.512
Intraoperative complication (yes)	1.903 (0.735)	0.010	1.384 (0.564)	0.279; 2.489	0.015	0.005 (0.160)	0.974	0.039 (0.165)	−0.285; 0.363	0.815

*ACD* anterior chamber depth,* AL* axial length, *CI* confidence interval, *SE* standard error, *WTW* white−to−white

## Association with CDVA

In this cohort, the maximum observed tilt and decentration along the CTA was 9.54° and 2.22 mm, respectively. Along the PA, the maximum values were 3.69° for tilt and 2.17 mm for decentration. Neither tilt nor decentration showed a detectable association with CDVA when adjusted for age, regardless of the reference axis used. For tilt along the PA, the unstandardized regression coefficient (B) was 0.004 (SE = 0.008), with a 95% confidence interval (CI) of − 0.011 to 0.020 (*P* = 0.592). For tilt along the CTA, B was − 0.006 (SE = 0.004), CI − 0.013 to 0.001 (*P* = 0.082). Regarding decentration, the B-coefficient for the PA axis was 0.012 (SE = 0.016), CI − 0.020 to 0.044 (*P* = 0.455), and for the CTA axis, 0.002 (SE = 0.018), CI − 0.034 to 0.037 (*P* = 0.925).

To evaluate whether the relationship between tilt or decentration and CDVA differed by IOL type, a second multivariable model was constructed including IOL type and the corresponding interaction terms. None of the interaction terms reached statistical significance: for tilt along the PA, B =–0.005 (SE = 0.018), CI − 0.039 to 0.030 (*P* = 0.790); for tilt along the CTA, B = 0.003 (SE = 0.008), CI − 0.069 to 0.092 (*P* = 0.717); for decentration along the PA, B = − 0.028 (SE = 0.035), CI − 0.098 to 0.042 (*P* = 0.430); and for decentration along the CTA, B=–0.052 (SE = 0.040), CI − 0.131 to 0.028 (*P* = 0.201). These findings indicate no significant effect modification by IOL type on the relationship between either tilt or decentration and CDVA.

## Discussion

This study evaluated the association between postoperative IOL tilt and decentration and CDVA in patients who underwent cataract surgery, providing valuable insights into their clinical implications. Using IOLMaster700 data and custom-developed software, our findings suggest that within the limited ranges of tilt and decentration observed, no detectable association with CDVA was found. The tilt values reached a maximum of 3.7° along the PA and 9.5° along the CTA, while mean decentration was 0.27 mm on both axes. In view of reported thresholds suggesting that tilt exceeding 5–7° or decentration beyond 0.4–0.5 mm can adversely affect visual outcomes, it is notable that the majority of eyes in our cohort remained below these levels [4–8]. This restricted range likely explains why no significant association with CDVA was detected in our study. Regression coefficients were close to zero, and the confidence intervals suggest that within the observed ranges, any potential association with CDVA is unlikely to be clinically meaningful. As the study was not powered for equivalence testing, possible effects in cases with larger tilt or decentration cannot be excluded.

Over the past decades, various methods for analyzing tilt and decentration have been studied, including Purkinje imaging, Scheimpflug imaging, ultrasound biomicroscopy, and anterior segment OCT (AS-OCT), the latter of which remains to be widely used. Among these, AS-OCT using SS-OCT provides high-resolution imaging and greater repeatability for tilt and decentration measurements compared to older techniques [21]. The IOLMaster700 and CASIA2 (Tomey Corp., Nagoya, Japan) are SS-OCT devices commonly used for such analyses. The IOLMaster700 uses custom software and different reference axes can be applied, whereas the CASIA2 employs built-in software for these analyses with the CTA as reference axis. Previous studies have demonstrated a high level of reproducibility of the CASIA2 for assessment of tilt and decentration, and excellent repeatability for tilt analyses of IOLMaster700 data utilizing custom software, using a similar methodology to our custom-developed software [22, 23]. This study is the first to analyze decentration with IOLMaster700 data and custom-developed software. By using a whole-eye scanning device, IOL positioning is assessed with reference to the fovea, with the patient fixating the optical axis during the imaging [2, 24]. However, while patient fixation is assumed to enhance measurement accuracy, it does not fully ensure alignment of the reference axis with the visual axis.

In this study, different reference axes were employed for tilt and decentration analyses. Although the PA and CTA are often used, there is uncertainty regarding which method best simulates the visual axis. The PA can easily be located but may vary with pupil shape, whereas the CTA is independent of pupil shape but more complex to determine. Furthermore, the literature indicates that there is tilt and decentration of the crystalline lens with respect to the corneal vertex [15–17, 25, 26]. Another approach for assessing tilt and decentration is the limbus-to-limbus method, which was not employed in this analysis [27]. Our results showed lower tilt outcomes along the PA compared to the CTA, while similar outcomes for decentration were found for both axes. A study using Scheimpflug imaging observes significant differences along the CTA and PA in tilt magnitude and decentration along the 180-degree meridian, but not along the 90-degree meridian. This discrepancy may be attributed to the nasal displacement of the corneal vertex relative to the pupillary center and may explain the differences in tilt outcomes between the PA and CTA in our study [15]. The similar decentration outcomes, which contradict previous findings, could be attributed to the use of Euclidean distances between horizontal and vertical outcomes in the study.

The literature identifies several variables associated with IOL tilt and decentration, yet the results can be influenced by the use of different devices and reference axes. Most studies focus on the CASIA2 and the CTA as reference axis. Significant positive associations with tilt have been reported for angles alpha and kappa, and previous PPV, whereas negative associations are reported for AL and ACD [3, 23–25, 28–30]. For decentration, AL and previous PPV show significant positive associations [3, 25, 29, 31]. One study has also found angles alpha and kappa, as well as age, to be positively associated with decentration [28]. 

Our analysis confirmed a significant positive association between angle alpha and IOL tilt along both axes, with other associations varying by reference axis. Combined surgery showed a significant negative association with tilt along the PA, whereas for tilt along the CTA positive associations were found with angle kappa and intraoperative complications, and a negative association with AL. However, as only six patients underwent combined surgery, no definitive conclusion can be drawn. Unlike previous studies, we found no association between previous intraocular surgery and tilt or decentration, possibly due to the small sample size. Intraoperative complications in this cohort, including three posterior capsular ruptures, were significantly associated with tilt along the CTA only. Further investigation with a larger sample size is required to assess these associations. For decentration, our analysis only identified a significant positive association with AL along both axes.

The impact of tilt and decentration on CDVA remains uncertain according to existing literature. One study reports that tilt < 4° and decentration < 1 mm along the PA do not significantly affect CDVA [17]. Conversely, other studies observes a significant correlation between CDVA, intermediate and near visual acuity and decentration towards the temporal and inferior quadrants when measured along the CTA [15, 32, 33]. In our cohort, tilt and decentration were not significantly associated with CDVA, with observed tilt values reaching up to 3.7° along the PA, and 9.5° along the CTA, and mean decentration outcomes of 0.27 mm along both axes. Even with a maximum decentration exceeding 2.00 mm, one patient still achieved a CDVA of 0.02 logMAR. These findings challenge the existing thresholds for clinically significant tilt and decentration, underlining their dependence on methods and reference axis used for analysis.

When interpreting our results, it is important to note that this cohort primarily consisted of patients with aspheric monofocal IOL designs, along with a group of non-diffractive EDOF IOLs. The literature indicates that the effects of tilt and decentration vary by IOL design [1, 5, 6]. Studies suggest that correct IOL positioning is particularly important for aspheric, toric, and multifocal IOLs, although reported findings are inconsistent [6, 34, 35]. A cohort study using adaptive optics has simulated the impact of tilt and decentration on different monofocal IOL designs, reporting a mean CDVA difference of 0.11 logMAR between centered and 0.2 mm decentered IOLs, and a 0.14 logMAR difference for 2° tilt. Aspheric IOL designs are found to be more sensitive to tilt and decentration with a limited compensating effect on spherical aberrations [36]. In contrast, a Scheimpflug imaging study finds no significant correlation between CDVA and IOL tilt or decentration in patients with monofocal or refractive mIOLs, with mean tilt ranging between 1.09 and 1.44°, and decentration ranging between 0.14 and 0.19 mm, respectively. However, the reference axis used in this study has not been specified [14]. Furthermore, a laboratory evaluation reports significant reductions in modulation transfer function (MTF) for monofocal IOLs only when decentrations exceed 0.75 mm [37]. Additionally, an optical bench study highlights the design-dependent variability of tilt and decentration effects on EDOF IOLs. The Tecnis Eyhance IOL (Johnson & Johnson Vision, Santa Ana, United States of America) shows a high sensitivity to tilt and decentration, while the Vivity (Alcon Laboratories Inc., Forth Worth, United States of America) and LuxSmart (Bausch & Lomb GmbH, Berlin Germany) IOL exhibit greater tolerance to tilt but are notably affected by decentration. The RayOne EMV (Rayner, Worthing, United Kingdom) demonstrates the highest robustness to tilt and decentration for small apertures but deteriorated with larger apertures, with decentration generally having a greater impact than tilt [38]. Another study suggests that a infero-temporal centration relative to the CTA in trifocal IOLs reduces the occurrence of photic phenomena, as measured by the Light Distortion Index [39]. These results emphasize the significant variability in sensitivity to tilt and decentration, driven by differences in IOL design and technology.

This study has several limitations, including a narrow range of tilt and decentration outcomes and a limited selection of IOL designs within the cohort. Orientation-dependent analyses were not conducted due to data constraints. However, we expect that such analyses would not have resulted in statistically significant differences in relation to CDVA. Patients unable to maintain proper fixation were excluded, as compromised fixation could diminish OCT scan quality, though pronounced tilt might itself impair fixation and contribute to selection bias. Additionally, heterogeneity in devices and reference axes used in previous studies complicates direct comparisons with our findings. While these limitations reduce the generalizability of our study, they emphasize the need for standardization of methodologies.

The absence of a statistically significant association between IOL tilt or decentration and CDVA in this cohort may be clinically reassuring, particularly for patients receiving standard aspheric or non-diffractive EDOF lenses. These findings suggest that, within the observed ranges of tilt and decentration, visual outcomes remain stable in an average-risk population. Given that the IOLMaster700 is widely used in clinical practice, our results provide relevant reference data for routine postoperative assessment. However, the generalizability of these findings is limited, particularly in relation to diffractive multifocal or other premium IOL designs, which may respond differently to variations in IOL positioning. Future studies in more diverse cohorts and lens types are therefore warranted.

In conclusion, tilt and decentration analyses conducted along both the PA and CTA revealed differences in outcomes based on the reference axis applied, which underscore the importance of considering specific devices and reference axes when comparing tilt and decentration outcomes, as the methods of analysis cannot be used interchangeably. Key variables associated with tilt varied depending on the reference axis, whereas decentration consistently demonstrated a positive association with AL along both axes. In this cohort of patients predominantly implanted with monofocal and non-diffractive EDOF lenses, no statistically significant association was found between tilt and decentration and CDVA. These findings may serve as a practical reference for evaluating postoperative IOL position in routine clinical practice.

## Supplementary Information

Below is the link to the electronic supplementary material.

Supplementary Material 1 (DOCX 1.23 MB)

## Declarations

### Data transparency

Upon reasonable request, the coding of our custom-developed software can be shared. Patient-level data cannot be made publicly available, as the dataset cannot be truly anonymized and thus remains personal data under the GDPR.
