# Supplementary material for: Association of intraocular lens tilt and decentration with visual acuity using SS-OCT-based analysis
Source: Graefes Arch Clin Exp Ophthalmol. 2025 Oct 16;263(12):3423–32. doi: 10.1007/s00417-025-06965-9 (PMC12886261; doi:10.1007/s00417-025-06965-9)
Supplement: Supplementary file 1 — Supplementary Material 1 (DOCX 1.23 MB) [file 417_2025_6965_MOESM1_ESM.pdf]

# Appendix 1. User Manual: Custom Software for Tilt and Decentration Analysis

Table of Contents

1 Installation ........ 1
1.1 MATLAB ........ 1
1.2 Runtime ........ 1
1.2.1 Separatly installed ........ 1
1.2.2 Complete installation ........ 1
2 Folder structure ........ 3
2.1 Example dataset ........ 3
2.2 Aggregated vs NotAggregated images ........ 4
2.3 Program folders ........ 4
2.3.1 SavedData ........ 4
2.3.2 ExcelOutput ........ 5
3 Running the program ........ 6
3.1 Select dataset ........ 6
3.1.1 Existing dataset ........ 7
3.2 User interface ........ 8
3.2.1 Manual Iris selection ........ 8
3.2.2 Manual cornea selection ........ 11
3.2.3 Manual lens selection ........ 11
3.2.4 Recalculate Tilt and Decentration ........ 12
3.2.5 Export to Excel ........ 12
3.2.6 Batch mode ........ 13
4 Method ........ 14
5 Improvements ........ 18
6 Bibliography ........ 19

# 1 Installation

There are multiple ways to run the program. These will be explained below. For the runtime versions of the program, there is a version with or without the command prompt. The version with the command prompt will return errors in case these happen. So if there needs to be any debugging using the standalone version then these should be used. Do note that the code itself cannot be changed for the standalone version as provided. This can be only used to see if the data is provided incorrectly or similar issues. Otherwise the MATLAB scripts have to be altered and compiled into standalone programs again. Furthermore, as the program has been compiled on a Windows PC it is only possible to run the program on a Windows system. Even running the MATLAB script can be problematic on different operating systems.

## 1.1 MATLAB

The first option is to run (and alter if needed) the MATLAB scripts itself. This will need a MATLAB installation and the version used to create the program is MATLAB 2021b. Newer or older versions can work, but this all depends on whether functions have changed between the different versions. To run the program a couple of toolboxes have to be installed however. These are the following toolboxes:

- Signal Processing Toolbox (version used: 8.7)
- Symbolic Math Toolbox (version used: 9.0)
- Image Processing Toolbox (version used: 11.4)
- Curve Fitting Toolbox (version used: 3.6)
- Global Optimization Toolbox (version used: 4.6)

## 1.2 Runtime

For people without MATLAB experience it is easier to use the standalone programs. These are ran using MATLAB runtime. As the standalone programs were compiled in MATLAB 2021b the required runtime version is “9.11”.

### 1.2.1 Separately installed

Runtime 9.11 can be separately downloaded and installed from the MathWorks website (https://nl.mathworks.com/products/compiler/matlab-runtime.html ). Once the runtime has been installed, the program can be started by double clicking the IOLMaster_Tilt_And_Decentration.exe file from the “RuntimeSeparatelyInstalled” folders that have been provided (see Figure 1).

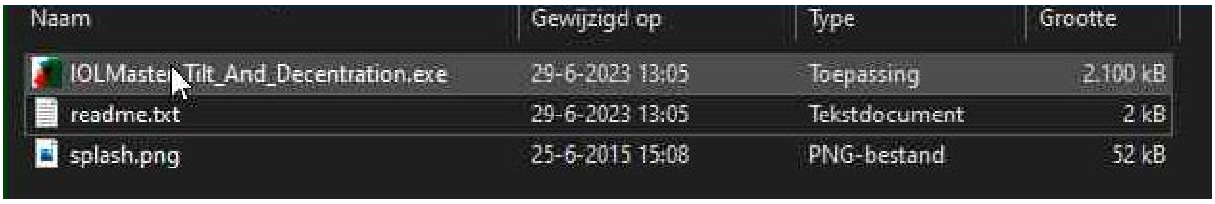


*Figure 1: Folder layout for the RuntimeSeparatelyInstalled folders. Double clicking the .exe file will run the program if the runtime has been installed.*

### 1.2.2 Complete installation

This process comes with one installer that will install the program on a user specified location (Default: C:\Program Files\IOLMaster_Tilt_And_Decentration). The program will also install the runtime that is required. The installation progress is straightforward and does not require any manual adjustments. After the installation has been completed, the program can be found at: “C:\Program

Files\IOLMaster_Tilt_And_Decentration\application\” when using the default location. This can be different depending on the system being used.

<u>Depending on the location where the program is stored, it may be necessary to run the IOLMaster_Tilt_And_Decentration.exe has to be ran as an administrator</u>. This is to ensure that the program can create folders or files when needed.

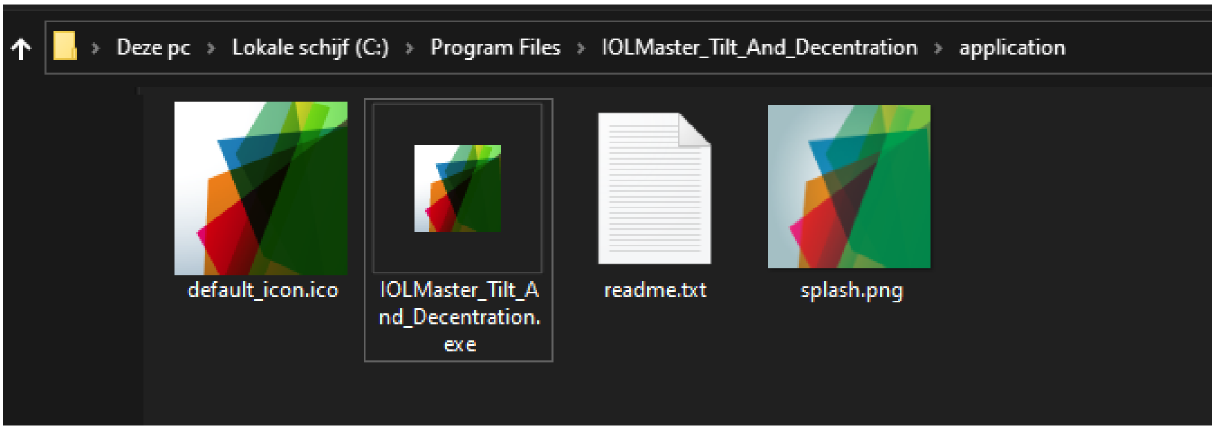


*Figure 2: Overview of the program location using the complete installation files. This is using the default location.*

## 2 Folder structure

For the tilt and decentration program to properly load and analyze the data the data should be structured in a way that is expected by the program. This structure will ensure that everything can be stored separately for each dataset and each measurement can be recognized within the program and the exported Excel files. To do this all the data, which belongs to one dataset, should be stored inside one folder. The name of this folder will be used in the program to see whether data has been processed already and to check whether any new data has been added to a dataset.

### 2.1 Example dataset

Within the dataset folder each measurement has a separate folder. This folder's name will be the name of the measurement within the program. To make sure that every measurement can be distinguished from one another all data for the measurement should be provided in the folder name. An example of this is shown in Figure 3, where the folder has been structured like: *"IDNumber_Eye_Date_Time"*.

| | | |
|---|---|---|
| 0001023718_OD_20220113_161522 | 29-6-2023 13:17 | Bestandsmap |
| 0001023718_OS_20220113_161522 | 29-6-2023 13:17 | Bestandsmap |
| 0001053030_OD_20220217_154731 | 29-6-2023 13:17 | Bestandsmap |
| 0001053030_OS_20220217_154731 | 29-6-2023 13:18 | Bestandsmap |
| 0001076954_OD_20220207_160539 | 29-6-2023 13:18 | Bestandsmap |

*Figure 3: Overview of the measurement folders with a dataset folder.*

Inside the measurement folder should be the exported data from the IOLMaster (see Figure 4). The folder, which is necessary for the analysis, is the AnteriorSegment folder. This contains the images which will be used to determine the tilt and decentration.

| | | | |
|---|---|---|---|
| AnteriorSegment | 29-6-2023 13:17 | Bestandsmap | |
| Configuration | 29-6-2023 13:17 | Bestandsmap | |
| Keratometry | 29-6-2023 13:17 | Bestandsmap | |
| Logs | 14-1-2022 11:34 | Bestandsmap | |
| Retina | 29-6-2023 13:17 | Bestandsmap | |
| RetinaAlignment | 29-6-2023 13:17 | Bestandsmap | |
| Sclera | 29-6-2023 13:17 | Bestandsmap | |
| BiometryData.csv | 13-1-2022 15:17 | CSV-bestand | 5 kB |
| PatientData.xml | 13-1-2022 15:16 | XML-bestand | 1 kB |
| Version.txt | 13-1-2022 15:17 | Tekstdocument | 1 kB |
| W2WImage.png | 13-1-2022 15:16 | PNG-bestand | 530 kB |

*Figure 4: Overview of the exported data that is inside the IOLMaster export.*

## 2.2 Aggregated vs NotAggregated images

The AnteriorSegment folder contains 12 images from two different types. Each type has six images each made at a different angle. The IOLMaster makes three measurements at each of the six angles and these are joined together to form the Aggregated images. The NotAggregated images are just one of the three measurements at that specific angle (see Figure 5).

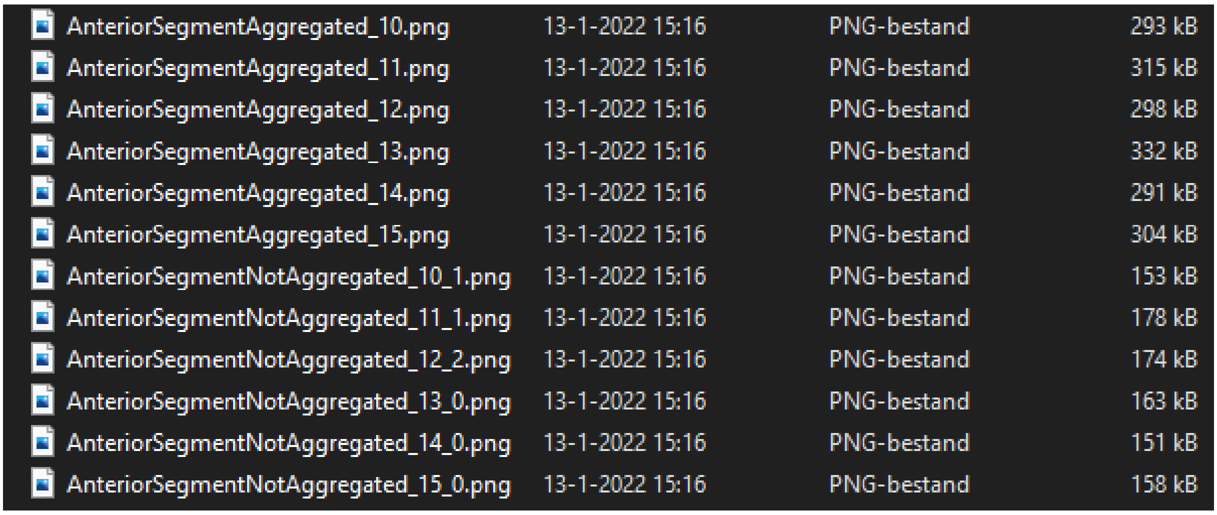

| | | | |
|---|---|---|---|
| AnteriorSegmentAggregated_10.png | 13-1-2022 15:16 | PNG-bestand | 293 kB |
| AnteriorSegmentAggregated_11.png | 13-1-2022 15:16 | PNG-bestand | 315 kB |
| AnteriorSegmentAggregated_12.png | 13-1-2022 15:16 | PNG-bestand | 298 kB |
| AnteriorSegmentAggregated_13.png | 13-1-2022 15:16 | PNG-bestand | 332 kB |
| AnteriorSegmentAggregated_14.png | 13-1-2022 15:16 | PNG-bestand | 291 kB |
| AnteriorSegmentAggregated_15.png | 13-1-2022 15:16 | PNG-bestand | 304 kB |
| AnteriorSegmentNotAggregated_10_1.png | 13-1-2022 15:16 | PNG-bestand | 153 kB |
| AnteriorSegmentNotAggregated_11_1.png | 13-1-2022 15:16 | PNG-bestand | 178 kB |
| AnteriorSegmentNotAggregated_12_2.png | 13-1-2022 15:16 | PNG-bestand | 174 kB |
| AnteriorSegmentNotAggregated_13_0.png | 13-1-2022 15:16 | PNG-bestand | 163 kB |
| AnteriorSegmentNotAggregated_14_0.png | 13-1-2022 15:16 | PNG-bestand | 151 kB |
| AnteriorSegmentNotAggregated_15_0.png | 13-1-2022 15:16 | PNG-bestand | 158 kB |

*Figure 5: Overview of the images that can be used for analysis.*

The standalone programs will look for the NotAggregated images inside the folder structure. This cannot be changed within the standalone programs. It can be changed within the MATLAB script however. This is done by commenting line 22 in readIOLMasterData.m and uncomment line 23. The dataset will then have to be analyzed again. In the case that the dataset has been analyzed and saved already this can be easily done by renaming the dataset folder within the SavedData folder, which is created by the program (see 2.3.1).

## 2.3 Program folders

The tilt and decentration program creates two folders which are used to store the processed data and exported excel files. These folders will be created (if needed) every time the program is started and will be created in the folder where the standalone program is stored or installed.

### 2.3.1 SavedData

Within the SavedData folder all processed data is stored. This is done in separate folders for each dataset. Within the folder for one dataset, each measurement has a separate .mat file. The SavedData folder is used to easily check processed data after the program has been shut down and keeps track of the measurements that have been processed already within a dataset. This is done by checking the name of the selected folder (see select dataset folder) and check if this already exists in the SavedData folder. If the dataset is already, present it will then check to see if there are any new measurements within the selected dataset folder. In the case that there are new measurements, the program will only analyze the missing measurements. The already analyzed measurements will be loaded from the SavedData folder when needed. In the case that there are measurements within the SavedData folder, which are not present in the selected data folder the program will not remove these and they will still be able to be viewed within the user interface (UI). In the case measurement have to be reprocessed, it is easiest to either delete the measurement's .mat file from the dataset's SavedData folder or to rename the dataset folder or the dataset's SavedData folder to analyze the entire dataset again.

### 2.3.2 ExcelOutput

All excel exports will be saved within this folder unless a different directory is selected during the export process.

# 3 Running the program

Running the program depends on the type of installation that has been done. This determines how to start the program. Once the program has been started, the functionalities are the same, besides some functionalities that are included within MATLAB itself. Within MATLAB, it is possible to pause and stop the program evaluation, but this will not be discussed within this manual. In case the program is ran from within MATLAB the main.m script should be ran. When using the standalone versions the IOLMaster_Tilt_And_Decentration.exe file should be executed to start the program. The exact location can differ depending on the installation or where the folder has been placed, but this is also briefly discussed in 1.2.1 and 1.2.2. The following section will continue from after the program has been started.

## 3.1 Select dataset

If the program is being ran as a standalone program the user will see a splash screen, which indicates that the program is starting up. This can take a while especially the first time after having turned on the computer. This is only part of the standalone program and is not part of the program itself. This splash screen will disappear automatically and no further user input is needed for the program itself to start as well. The tilt and decentration program itself does need the user to provide the location of the dataset that has to be analyzed.

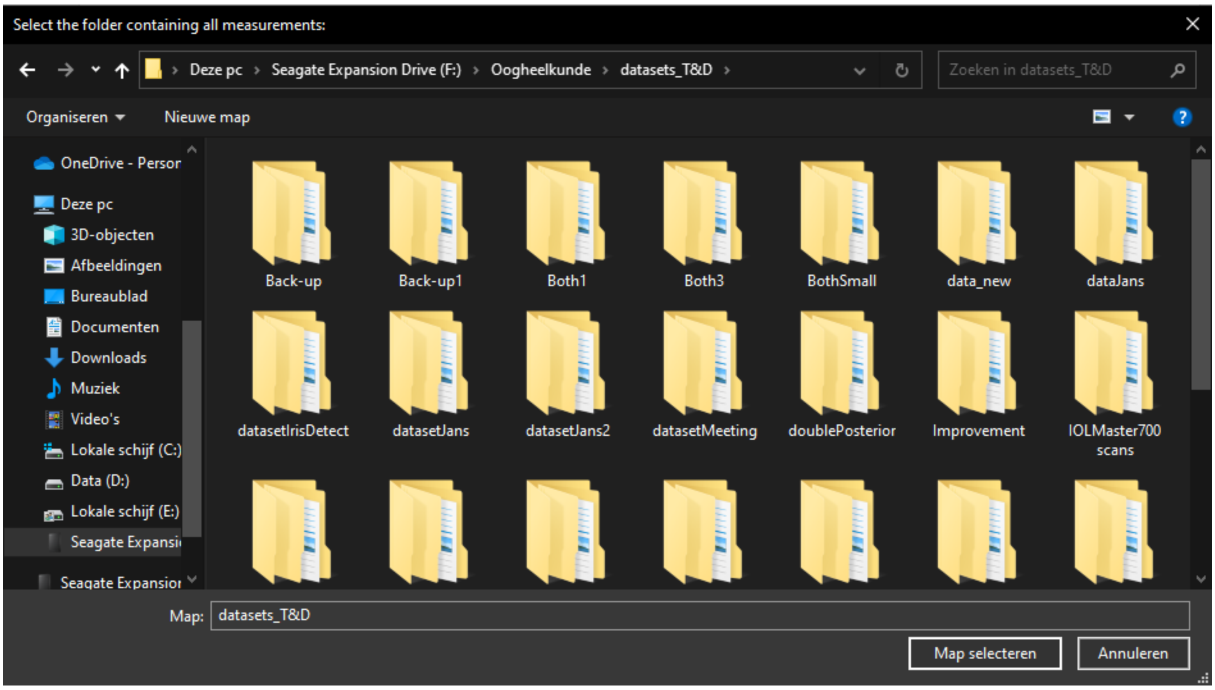


*Figure 6: Pop-up from the program that asks the user to select a dataset folder. This folder should contain folders with one measurement's exported data within.*

After selecting a folder, the program will determine the amount of measurements that have to be imported and analyzed. Afterwards a pop up will show up which will display the progress of the analysis. Do not close this pop-up window, as the program will crash after closing it.

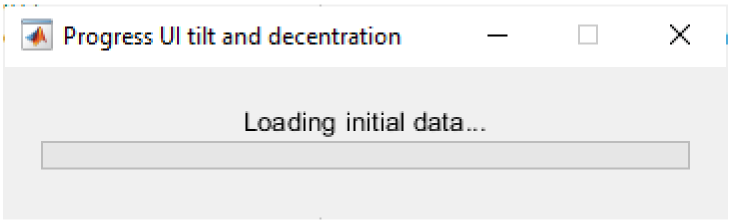


*Figure 7: Pop-up to indicate the progress of the program.*

After selecting the folder the program does not need any further user input. However, it is possible that the standalone versions will ask for permissions when being ran for the first time. After the first slice of the first measurement has been completed, the program should run without asking any user input. The amount of time necessary to analyze the dataset depends on multiple factor. However, during testing it took roughly 6 minutes for every measurement in the dataset. Once the analysis has been completed, the program will open the UI, which can be used to review, adjust and export the results.

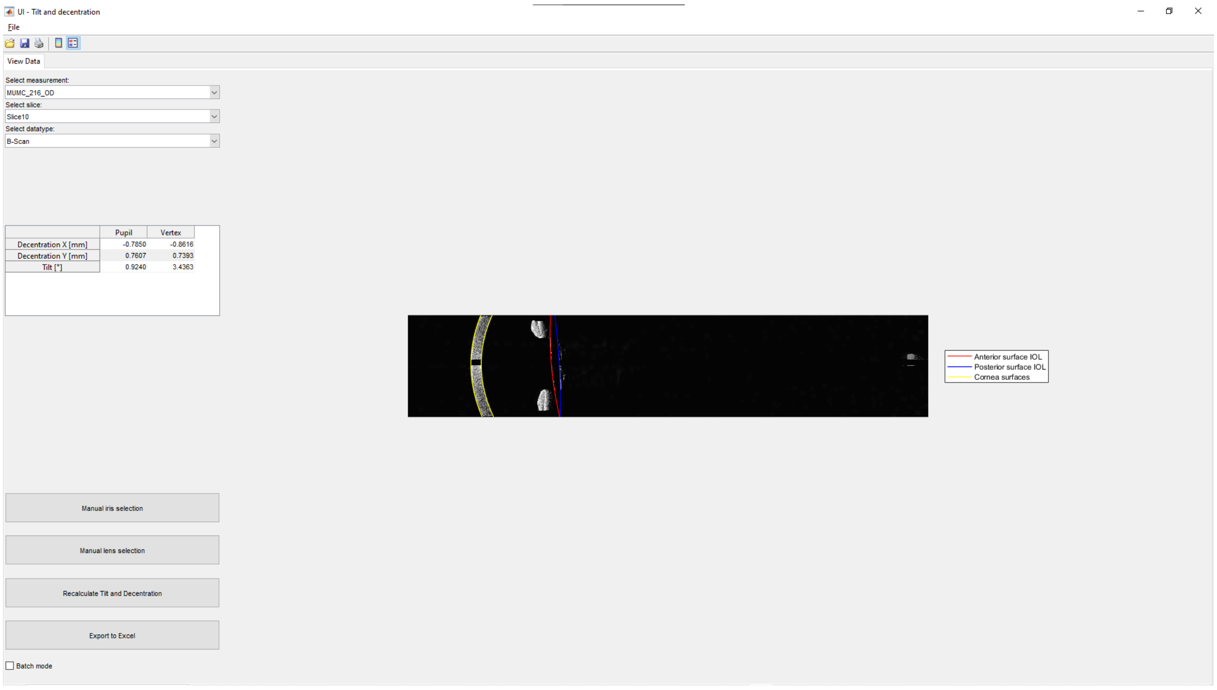


*Figure 8: User interface that will be launched after completing the dataset analysis.*

### 3.1.1 Existing dataset

If a dataset has been analyzed already it can be reviewed again by selecting the dataset folder again. If any new measurements have been added these will be analyzed and the progress pop-up will be shown as before. In the case there are no new measurements the progress pop-up will show that the analysis has been completed and the UI will be launched afterwards.

In the case an existing dataset (inside the SavedData folder) is to be reviewed but the dataset folder itself is not accessible, the data can still be reviewed by creating an empty folder with the name of the dataset. In this case the program will have no new data to analyze and opens the UI with the data that was already stored.

## 3.2 User interface

The UI consists of different section each with their own functionality. The dropdown menus in the top left are used to browse through the measurements and the individual slices. The bottom dropdown menu is used to swap the type of data that is being viewed. The b-scans are usually are enough to make the manual adjustments. However, the segmentation will show the cornea, iris and lens separately and is therefore useful to check if these have been selected properly. The 3D plot option shows the data organized into a three-dimensional coordinate system. The origin of this coordinate system is the center of the pupil at every one of the six slices and the angle at which each slice is taken determines their orientation within the coordinate system. The plane fit plot shows the plane which has been fitted through the lines indicating the middle of the lens (line through intersections of the front and back lens surface). The tilt plot shows the two axis over which the lens tilt is calculated and shows the central lens plane and its normal vector. This normal vector is an arrow perpendicular to the plane, which is used to calculate the angle of the plane. The thickness plots show the thickness of the lens and this is combined into 3D in the thickest point plot. In the thickest point plot, the individual slice thicknesses are combined by fitting a paraboloid to estimate the thickness of the lens in 3D.

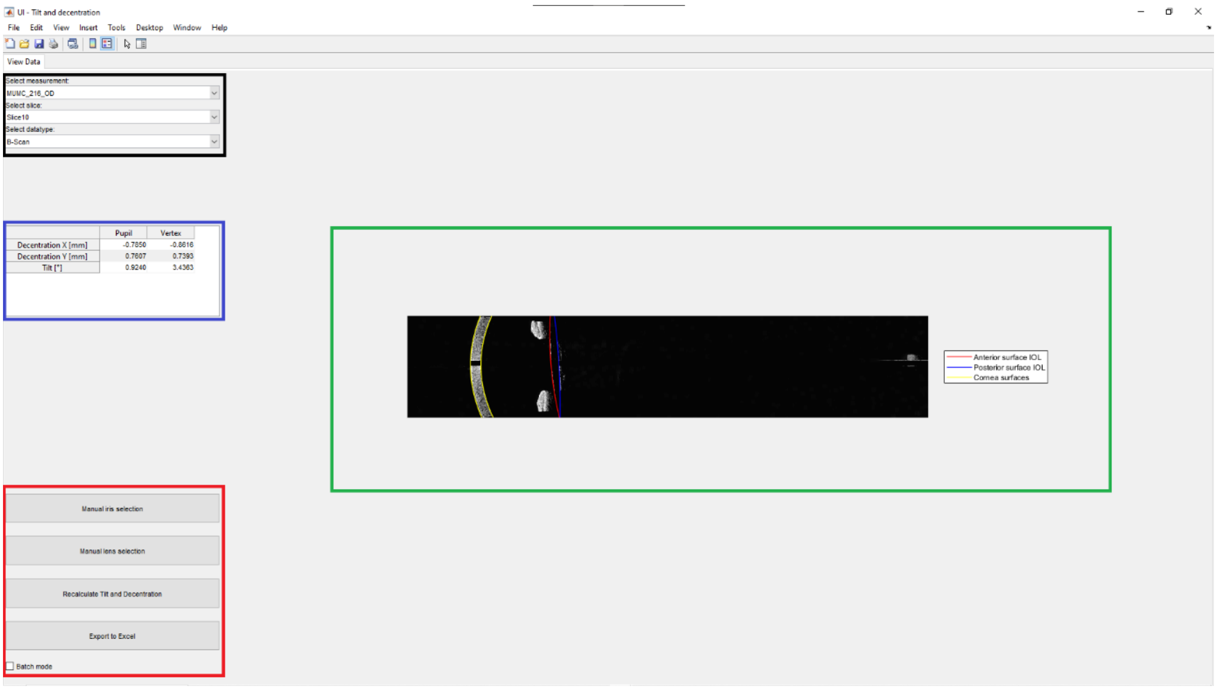


*Figure 9: Overview of the content in the UI. The dropdown menus in the black rectangle are used to navigate through the dataset's measurements. The table in the blue rectangle shows the calculated parameters for the current measurement. The buttons inside the red rectangle are used to manually change the current slice of the measurement, to recalculate the entire measurement or to export all measurements in the dataset to Excel. Inside the green box is where the UI will display the data of the current measurement. This will vary depending on the selected datatype however.*

Besides viewing the measurements, it is also possible to manually or semi-automatically change the measurements in case the original analysis is incorrect.

### 3.2.1 Manual Iris selection

Pressing the manual iris selection button will open a pop up which is used to make all the changes to the iris detection and cornea fit (see Figure 10). When making manual changes first it is important to check if the checkboxes are correct. In the case both the upper and lower iris are visible in the B-scan then the corresponding checkbox should be ticked. In the case the B-scan contains a natural lens the natural lens checkbox should be checked.

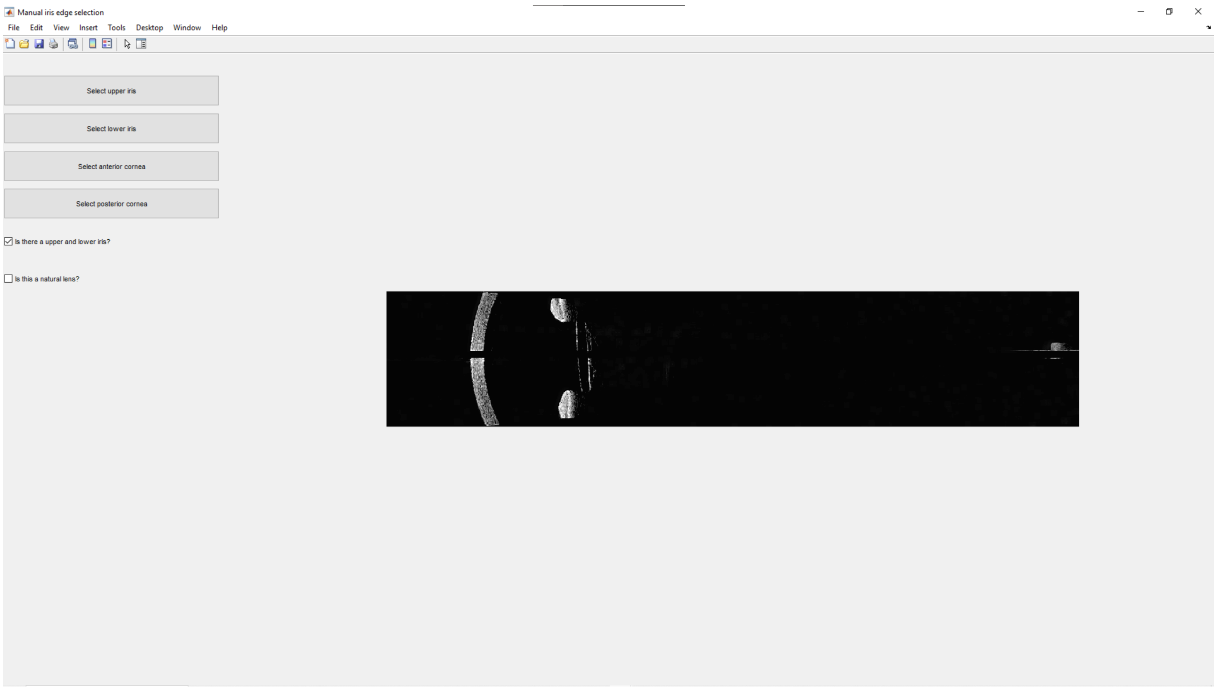


*Figure 10: The iris and cornea pop-up interface. The buttons on the left enable the user to place points in the image to the right. The checkboxes can be toggled to give information about the image.*

To make manual changes to the corresponding iris button should be pressed (Select upper iris/Select lower iris). The other buttons will now grey out and cannot be used until the user finishes the iris selection. The functionality of the iris selection depends on the type of lens in the measurement. When the lens is an IOL, the user simply has to click inside the iris when the cursor looks like a crosshair (see Figure 10 and Figure 11). For optimal accuracy, it is recommended to click at the center of the iris, since the coordinates are subject to rounding." If the scan contains a natural lens however the user should locate the point on the edge between the iris and the natural lens (see Figure 12). This is because the program will use this point to separate the lens from the iris using a diagonal line. The angle of the diagonal line is limited however and will therefore never be horizontal or vertical.

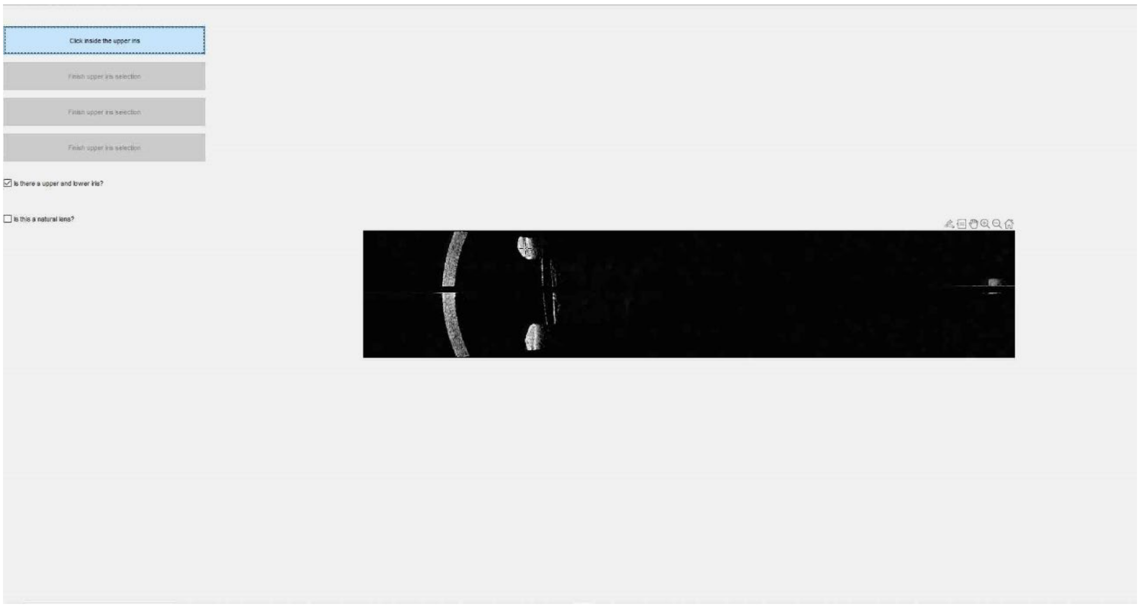


*Figure 11: After clicking either the upper or lower iris button the cursor will change to a reticle when hovering over the image. This can been seen in the image above by zooming in on the upper iris.*

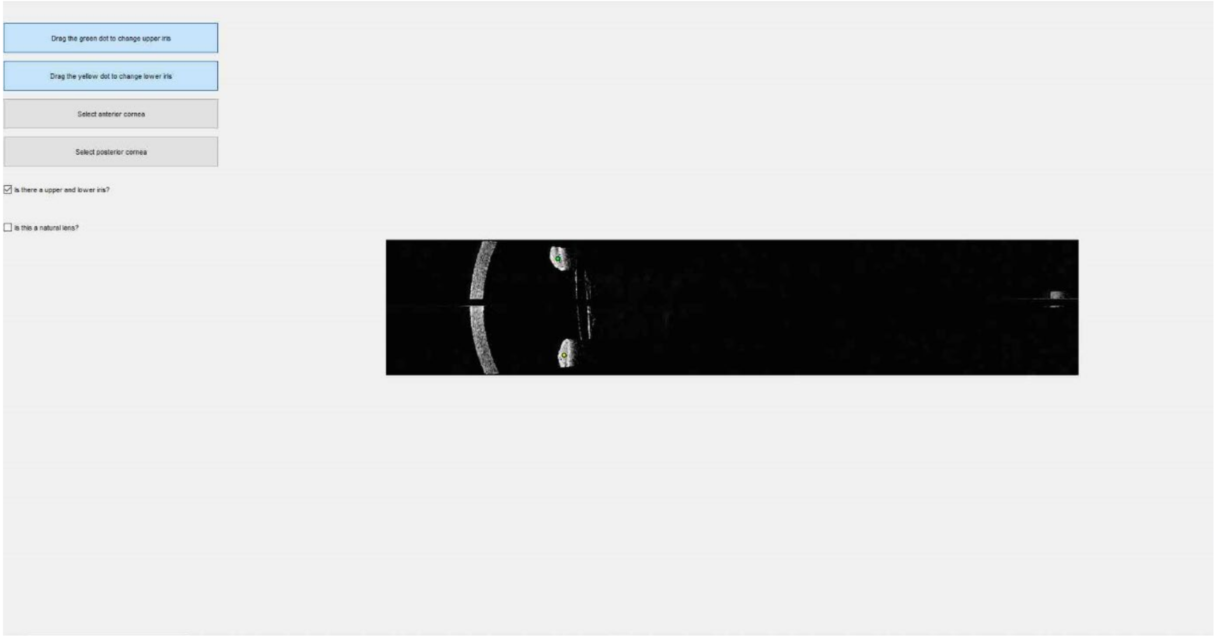


*Figure 12: after selecting the iris position, a dot will be placed on the spot that was selected. This dot can be dragged by holding the left mouse button and dragging the mouse to adjust the location of the dot.*

It is also possible to adjust the cornea fit using this interface this will be discussed in section. These adjustments can be done within the same instance of the iris/cornea manual adjustment procedure. Once the manual changes have been completed the pop up can be closed and the program will start to recalculate the data using the new manual adjustments.

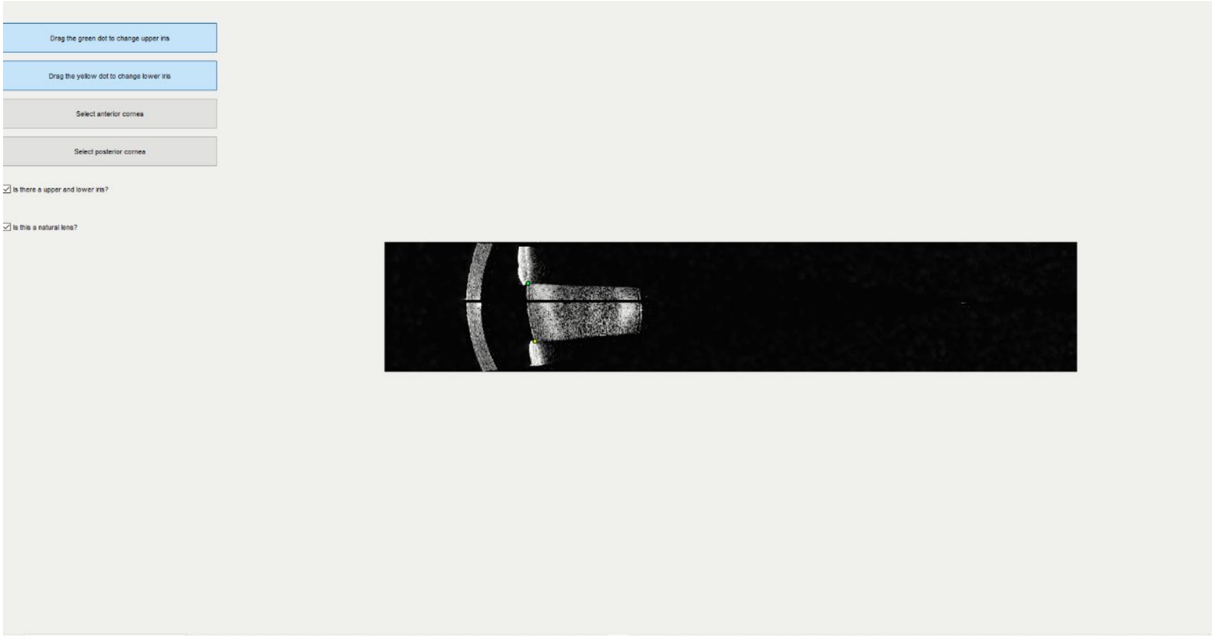


*Figure 13: Placement for the split off for the iris selection in case of a natural lens. As can be seen when zooming in on the image the dots are located in between the iris and the lens. The green dot represents the edge between the upper iris and the lens and the yellow dot the edge between the lower iris and the lens.*

### 3.2.2 Manual cornea selection

The manual cornea selection is within the same pop up as the iris selection. This is opened by pressing the "Manual iris selection" button in Figure 9. This will open a pop up window in which the cornea fit changes can be made. Using the "select anterior/posterior cornea" buttons the user can determine where the anterior and posterior surface of the cornea is located in the image. This is done by selecting 3 points along the selected surface of the cornea (see Figure 14). Optimal point placement involves positioning two points at the corneal periphery and one near the center. This should be the best indication of the overall shape of the cornea. As this is a semi-automatic process, the points do not have to be exactly on the corneal surface. The program only uses the user input as a guideline and determines the best fit close to the input of the user. Once the user has clicked the select anterior/posterior cornea button, the other buttons will become inactive until the user has selected three points. Be aware that the program does not check whether the anterior surface is indeed in front of the posterior surface so make sure that the dots are placed correctly. The red dots represent the anterior corneal surface and the blue point the posterior surface. Once the dots are correctly placed, the pop-up window can be closed. This will start the data recalculation.

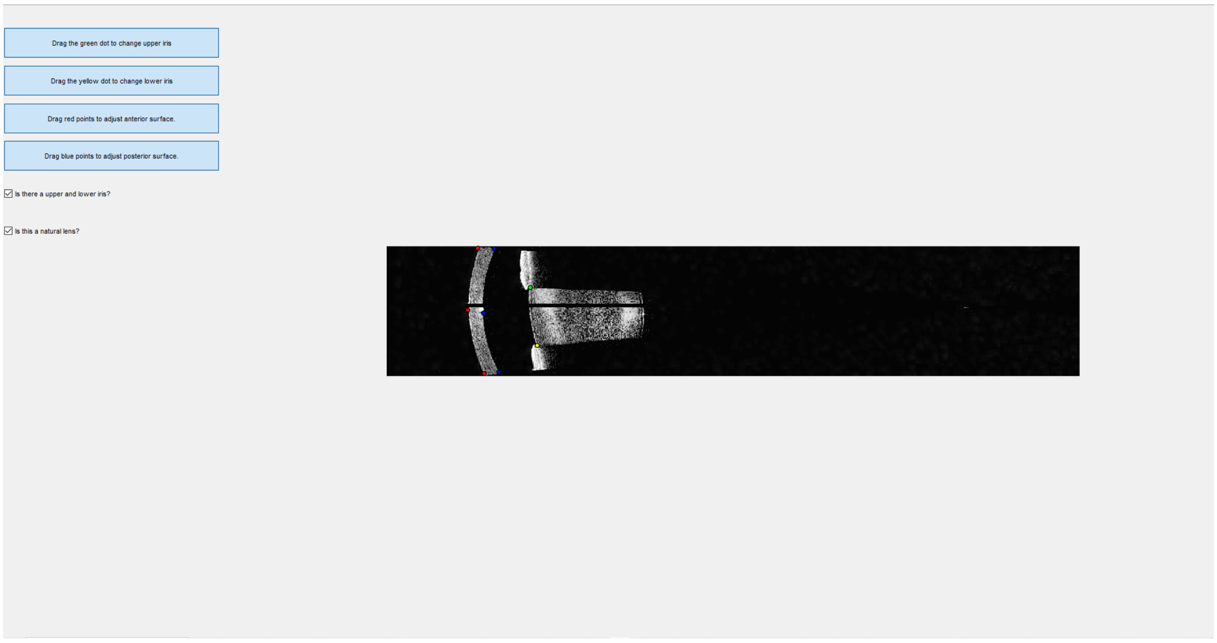


*Figure 14: Manual selection of the corneal surfaces. The points (for each surface) are spread out as much as possible to give an indication of the entire surface. Both the iris selection and the corneal surface selection have been completed in this example and these will both be used in the recalculation once the pop-up window is closed.*

### 3.2.3 Manual lens selection

When the manual lens selection button is pressed, the corresponding pop-up will open. For the lens selection, both surfaces are assumed convex. This means that if the lens surface is concave the program will most likely do a bad fit, unless it is very close to being flat. In the case the program cannot do the lens fit correctly or the lens is barely visible the slice can be skipped from the calculations. This can be done by unticking the "Is the entire lens visible?" checkbox (no checkmark) and closing the pop-up window. If you want the slice to be manually adjusted, make sure that this checkbox is ticked. The process of selecting the lens surface is similar to cornea selection as the user has to select three points on the lens and the program will use this information to select the best fit near these parameters. The selected points do not necessarily have to be on the lens in the image. This means the points should mainly follow the trend of the lens. The three lens surface points are ideally placed as far apart as

possible. This is the case because the exact location of the dot does not influence the fitted parabola as much if the dots are further apart.

Sometimes the program can run into an error when fitting the lens surface. This tends to happen when the lens surface is nearly vertical or concave instead of convex. In this case, the progress pop-up window can be closed and the manual lens selection can be started again.

Besides selecting the anterior and posterior lens surface, it is also possible to create a division between the anterior and posterior lens surface. Especially in lenses which are rotated a lot in the B-scan the program can have issues with determining which pixels belong to the anterior and which belong to the posterior surface. By drawing a line in between the two surfaces the program will classify the pixels left of the line as anterior surface pixels and to the right for the posterior surface. Besides correctly classifying between the anterior and posterior surface the division line will not remove as much small components within the image. Therefore it can improve the lens fit if the lens surface consists of many small separated groups of brighter pixels. Once the manual adjustments have been completed, the pop-up can be closed to start the recalculation of the slice.

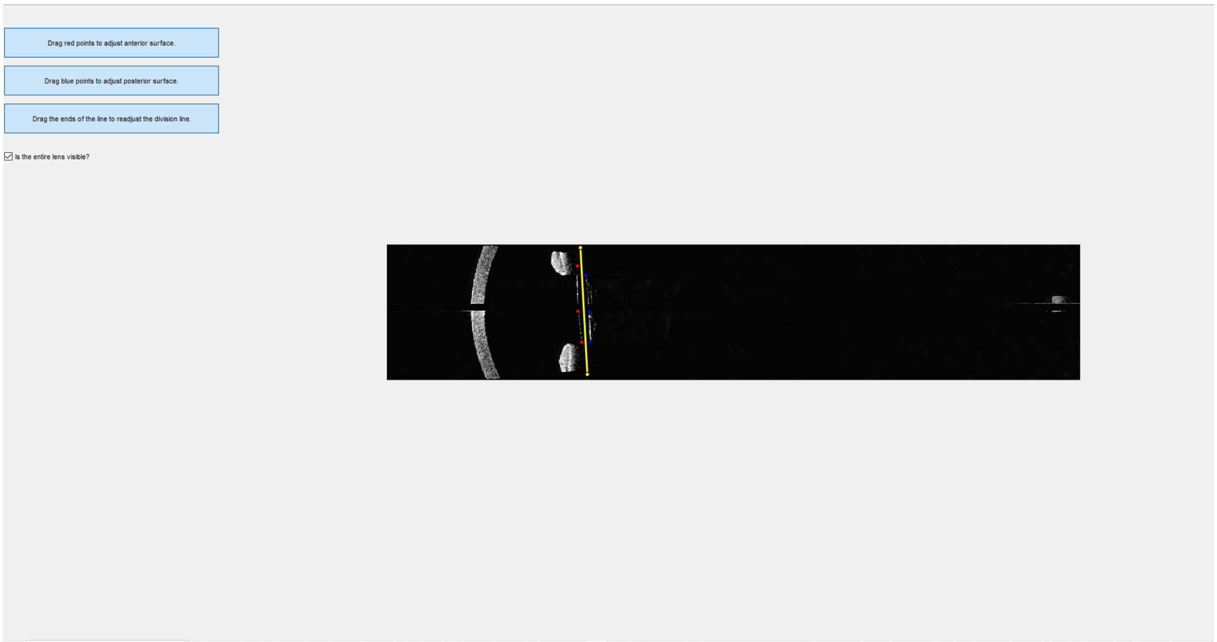


*Figure 15: Overview of the manual lens selection pop-up. The red dots represent the anterior lens surface and the blue dots the posterior lens surface. The yellow line is placed between the lens surfaces so the program knows exactly which pixels (in the image) belong to the anterior and posterior surface.*

### 3.2.4 Recalculate Tilt and Decentration

This button is used to recalculate the tilt and decentration once manual changes have been made. As the tilt and decentration are calculated from the 3D representation of the data this should be done after every slice of the measurement has been manually changed or checked to make sure the program has done the analysis correctly. After pressing the button, the program will show a pop-up to show the analysis has started. Once it has finished the message in this pop-up will change and after a couple of seconds, the pop-up will disappear. The user interface is updated to display the newly calculated values.

### 3.2.5 Export to Excel

This will create an excel file with the tilt and decentration values for every measurement in the dataset. The user can select the name and location for the excel file.

### 3.2.6 Batch mode

The batch mode checkbox will start the batch mode analysis. In this case, the manual changes are stored, but the recalculation will not take place. This will happen after the user interface has been closed. This mode does not always work well however as the recalculation is stopped when running into an error. This would mean that any manual changes after the error will not be recalculated.

# 4 Method

The tilt and decentration program starts by reading all necessary data for each measurement and makes sure that the b-scan is a grayscale image. The image is also cropped to reduce the amount of data that is stored. This speeds up analysis and removes a mostly black area of the image, which is beyond the retina in the b-scan. Therefore, no data should be lost by cropping the image. After loading in the data the b-scan analysis starts. The exact analysis depends on whether the b-scan contains a natural lens or an IOL. To determine this the program counts the amount of non-black pixels in each row of the b-scan. Afterwards the average amount of pixels in a row and the standard deviation is calculated. The mean amount of pixels in a row determines whether the b-scan is classified as a natural lens or an IOL. The threshold for this is set to a value of 170, which means a mean below or equal to 170 is classified as an IOL measurement and above is a natural lens. After this, the program will remove horizontal lines made from the b-scan. This is determined using the standard deviation on the mean amount of bright pixels in each row. Any row with more bright pixels than the mean + 1.3*standard deviation will be set entirely to zero. Afterwards Hough transform is used to look for diagonal and smaller horizontal lines which can be left in the image and if found these pixels will be set to zero as well. Afterwards the noise is removed as much as possible using non-local mean image denoising (Wu, 2012). Next, the b-scans are sharpened and contrast is increased.

Now that the image is pre-processed, the program will determine the rough location of the cornea, iris and lens and the rest of the image. This is done so that these parts can be binarized separately. This is done by calculating the Yen threshold for each of these parts separately and combining them back into the complete b-scan, resulting in a binarized b-scan. To remove the remaining noise all connected pixels consisting of less than 15 pixels. Afterwards the program detects the iris in the b-scan and determines the center of the pupil between the upper half and lower half of the iris in the image. This point is used as the origin for the slice when all six slices are combined in a 3D representation based on the orientation and angle for every separate slice. The pupil center is therefore used to line up the measurements together. In the case the iris halves in the b-scan are not visible or are too small (smaller than 100 pixels in size) then the slice will not be analyzed further as the slices cannot be combined into 3D. After the iris has been detected, the program will then fit parabolas with the formula $a(x - x_0)^2 + c$. These are fitted to the anterior and posterior edges of the binarized cornea in such a way that the parabolas go through as many white edge pixels as possible. Using the $x_0$ and c parameters, the vertex of the anterior cornea is determined. This will later be used to calculate the tilt and decentration.

Afterwards the IOL or natural lens are also fitted with parabolas with the function $a(x - x_0)^2 + c$. In the case of an IOL, the parabolas are fitted on the front of both the anterior and posterior surface and again the parabola should go through as many pixels as possible. However as the IOL and natural lens are multiple pixels thick, the best fit does not necessarily go through the most pixels. Therefore multiple weighted parabolas are added into the cost function, which will be minimized. The parabolas only differ in the value of the c parameter (see Figure 16). The parabola with the value c is the parabola that is used as the best fit and is also weighted the heaviest. Any parabola to the left (c-1 and so on) will increase the score and therefore make the fit worse. When the parabolas to the right (c+1 and so on) go through any pixels this will decrease the score but less compared to the original parabola due to the weight value. This same method is used for the natural lens only that in the posterior natural lens surface any parabola to the left (c-1 and so on) decreases the cost score and to the right (c+1 and so on) increase the cost score.

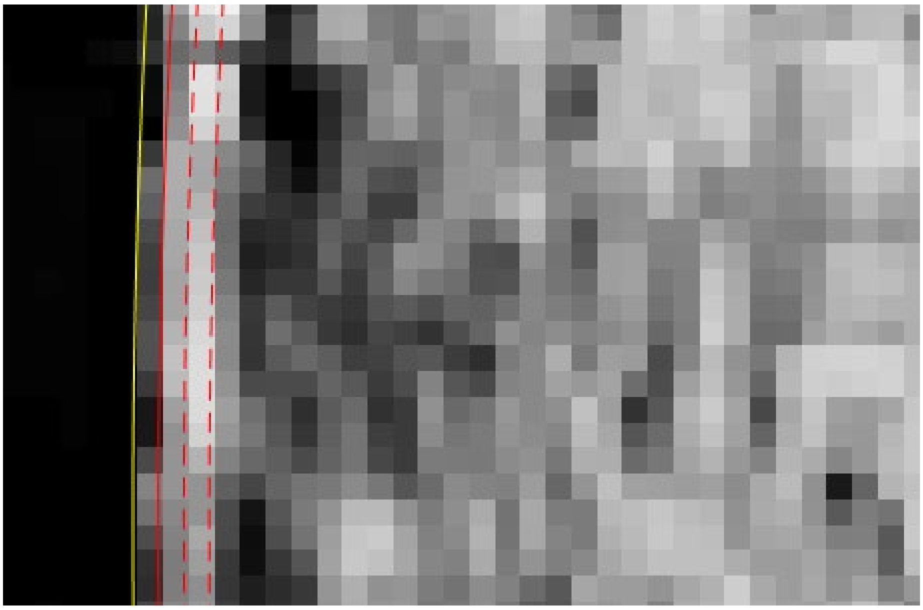

*Figure 16: Visual representation of the parabola fitting procedure. The red line is the parabola that has been fitted. The red dashed lines will improve (reduce) the fit cost if they go through pixels belonging to the lens. The yellow line should go through as few lens pixels as possible because it will increase the cost value.*

Once the lens surfaces have been fitted, the intersections between these parabolas are calculated and the line connecting these points is determined. This line is assumed to be going through the center of the lens. This centerline is calculated for each of the six slices and are then used to calculate the central plane of the lens in 3D. For each of the center lines 100 data points are calculated in the 3D reconstruction. Then a plane is fitted to these 600 data points resulting in the central plane of the 3D lens (see Figure 17). The normal vector of this plane is calculated to determine the lens tilt.

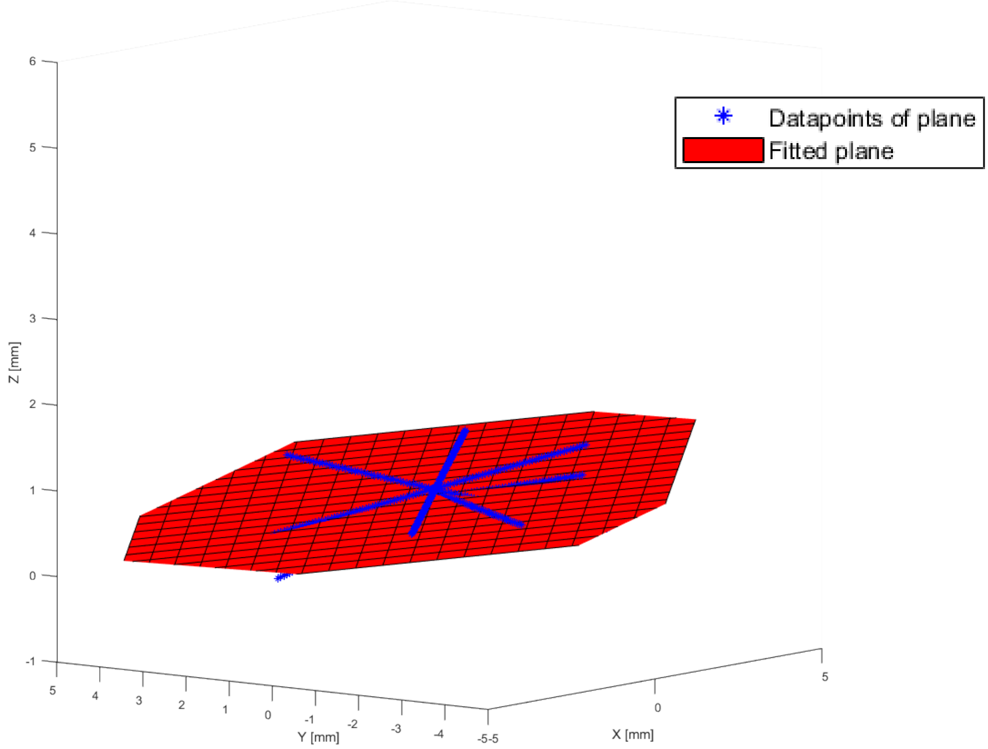


*Figure 17: Fitting the central lens plane to the data point from the centerlines from the individual slices.*

The lens tilt is determined against two axis. The first is the line perpendicular to the tangent going through the vertex of the anterior corneal surface. To determine an axis a paraboloid is fitted through the anterior corneal parabolas and afterwards the tangent plane is determined. The normal vector of this plane is the axis, which is used to calculate the lens tilt. The other axis is determined by using the line between the iris centers from each slice. Again, 100 data points are evenly distributed between these two points for all the six slices. From these 600 points, a plane is fitted to get the iris orientation from within all the six slices. The normal of this plane is then used as the other axis to calculate the lens tilt.

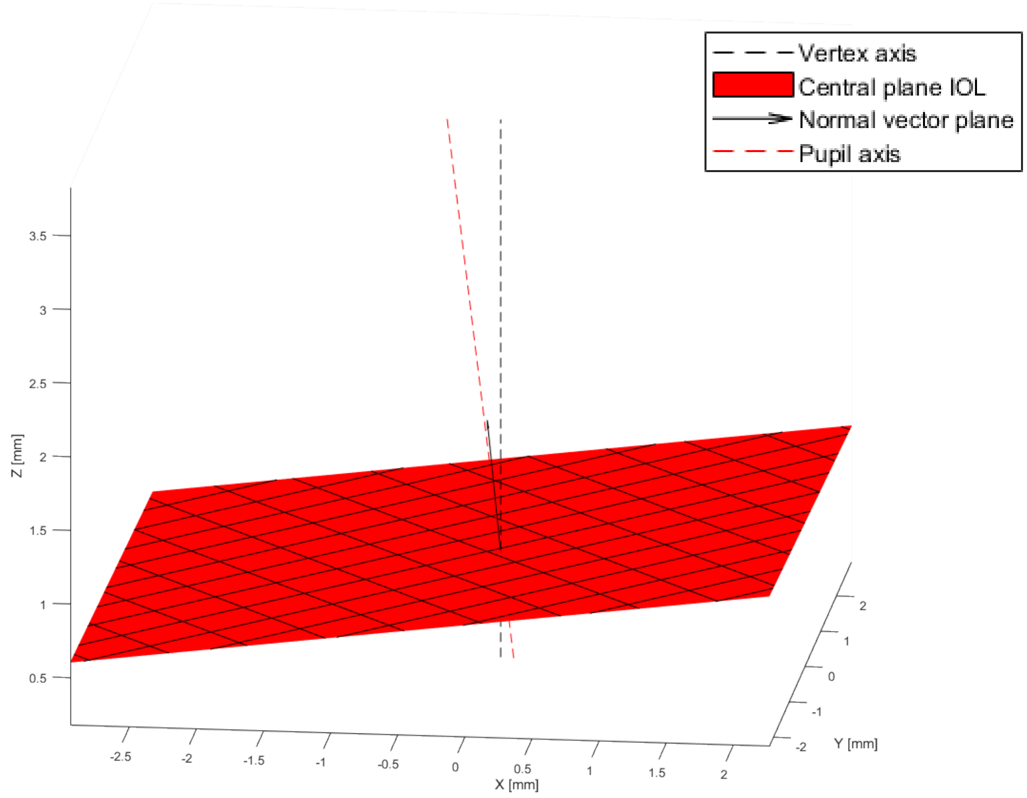


*Figure 18: Overview of the vertex and pupil/iris axis together with the normal vector of the central lens plane.*

To calculate the decentration of the lens it is necessary to determine the center of the lens. We have assumed the center of the lens to be the thickest point of the lens. Therefore the thickness of the lens in every slice was calculated. We have defined the lens thickness to be the distance between the anterior and posterior lens surface perpendicular to the line going through the center of the lens (see Figure 19). The thickness was then calculated at 100 evenly spread out points along this central lens line and a parabola is fitted to these 100 points for every slice.

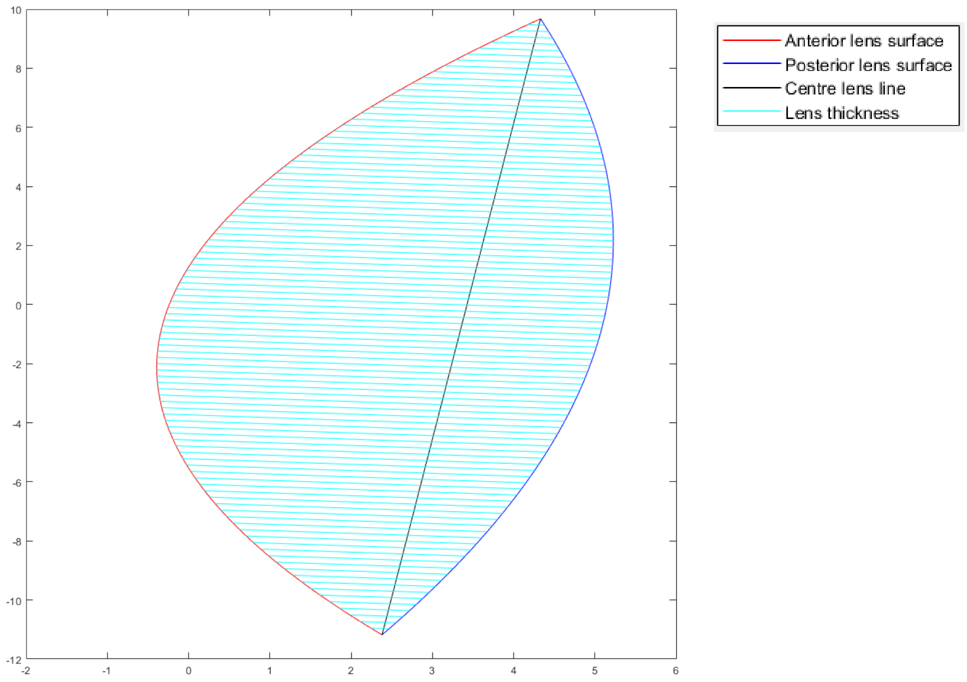


*Figure 19: Visual representation of the lens thickness calculation for a natural lens. The length of each line is equal to the calculated thickness.*

All the 600 thickness data points are also transferred into a 3D space to calculate the thickest point by fitting a paraboloid to the data points (see Figure 20). The formula for this paraboloid is: $a((x - x_0)^2 + (y - y_0)^2) + c$. The thickest point is then ($x_0$,$y_0$,c). To determine the decentration the same axis are used as the ones that have been used for the tilt. The decentration is calculated by determining the intersections between the two axis and the central lens plane. The difference in x and y coordinate between the two axis' intersection with central lens plane and the thickest point is then decentration of the lens compared to the axis.

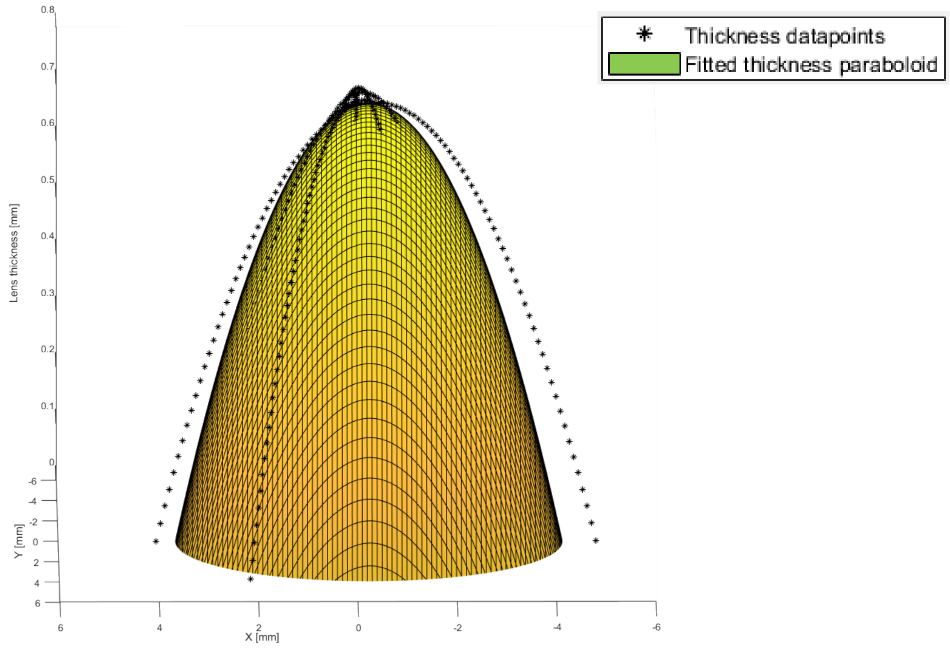


*Figure 20: Paraboloid fit on the thickness data points.*

## 5 Improvements

The first improvement which could be made is to change the origin of the 3D coordinate system to the vertex of the anterior cornea. This way the center and decentration can still be determined according to the vertex axis even if the iris is only partially or not visible in the b-scan.

The second improvement which can be made is by improving the paraboloid fit for the thickest point. In the current state, this paraboloid seems to be shifted too much because not all the slices line up around the same point. This misalignment could be due to a difference between the pupil centers between the measurements that would then result in everything else being misaligned.

## 6 Bibliography

Wu, Y. (2012, 09 18). *Fast Non-Local Mean Image Denoising Implementation.* Retrieved 07 14, 2023, from MathWorks: https://nl.mathworks.com/matlabcentral/fileexchange/38200-fast-non-local-mean-image-denoising-implementation
